# Thoracolumbar interfascial plane block for postoperative analgesia in spine surgery: A systematic review and meta-analysis

**DOI:** 10.1371/journal.pone.0251980

**Published:** 2021-05-21

**Authors:** Yu Ye, Yaodan Bi, Jun Ma, Bin Liu

**Affiliations:** Department of Anesthesiology, West China Hospital, Sichuan University, Chengdu, Sichuan, China; Cleveland Clinic, UNITED STATES

## Abstract

**Introduction:**

Thoracolumbar interfascial plane (TLIP) block has been discussed widely in spine surgery. The aim of our study is to evaluate analgesic efficacy and safety of TLIP block in spine surgery.

**Method:**

We performed a quantitative systematic review. Randomized controlled trials that compared TLIP block to non-block care or wound infiltration for patients undergoing spine surgery and took the pain or morphine consumption as a primary or secondary outcome were included. The primary outcome was cumulative opioid consumption during 0-24-hour. Secondary outcomes included postoperative pain intensity, rescue analgesia requirement, and adverse events.

**Result:**

9 randomized controlled trials with 539 patients were included for analysis. Compared with non-block care, TLIP block was effective to decrease the opioid consumption (WMD -16.00; 95%CI -19.19, -12.81; p<0.001; I^2^ = 71.6%) for the first 24 hours after the surgery. TLIP block significantly reduced postoperative pain intensity at rest or movement at various time points compared with non-block care, and reduced rescue analgesia requirement ((RR 0.47; 95%CI 0.30, 0.74; p = 0.001; I^2^ = 0.0%) and postoperative nausea and vomiting (RR 0.58; 95%CI 0.39, 0.86; p = 0.006; I^2^ = 25.1%). Besides, TLIP block is superior to wound infiltration in terms of opioid consumption (WMD -17.23, 95%CI -21.62, -12.86; p<0.001; I^2^ = 63.8%), and the postoperative pain intensity at rest was comparable between TLIP block and wound infiltration.

**Conclusion:**

TLIP block improved analgesic efficacy in spine surgery compared with non-block care. Furthermore, current literature supported the TLIP block was superior to wound infiltration in terms of opioid consumption.

## Introduction

Patients undergoing spinal surgery could suffer diffuse and severe postoperative pain. inefficient pain control after the surgery weakens the rehabilitation, prolongs the hospital stay, worsens patient satisfaction, and promote the development of persistent postsurgical pain [1–3]. Although plenty of pharmacological options such as gabapentin, nonsteroidal anti-inflammatory drugs (NSAIDS), and ketamine could be applied to reduce the opioid consumption in the multimodal analgesia for spine surgery, regional anesthesia techniques such as wound infiltration and thoracolumbar interfascial plane block are the cornerstone of postoperative pain management for spine surgery [4–6]. The role of wound infiltration in postoperative analgesia in spine surgery has been discussed widely. Thoracolumbar interfascial block (TLIP block) is a novel regional anesthesia technique first described by Hand et al. in 2015 [7], which is an interfascial plane block applied at the L3 vertebral level in spine surgery. Considering the potential risk of neuraxial injury and the difficulty in sonographic imaging of TLIP block, the modified TLIP block was described by Ueshima et al. in 2016 [8], which could provide comparable analgesia and opioid-sparing effect as the classic approach [9].

Recently, several studies have compared the novel techniques that target dorsal rami of the thoracolumbar nerves to either non-block care or other regional blocks in spine surgery [10–12]. However, many of these studies have resulted in contradictory findings. A quantitative analysis focusing on the efficacy and safety of these novel anesthesia techniques has not yet been performed.

Therefore, we decided to perform a systematic review and meta-analysis to compare the analgesic efficacy of TLIP block and/or modified TLIP block to non-block care or wound infiltration respectively in patients undergoing spine surgery. we also aimed to summarize complications associated with these regional anesthesia techniques.

## Method

We searched PubMed, Medline, Embase database, and Google scholar (between January 1985 and September 2020) using the following terms: “lumbar spine surgery”, “decompression”, “lumbar spinal stenosis”, “spondylolisthesis”, “thoracolumbar interfascial plane block” and “TLIP block”. We also searched the gray literature by supplementary hand searching for the TLIP block being a new regional anesthesia technique firstly introduced in 2015. Language restrictions were not used. There was no limitation on sample size.

Inclusion criteria were as follows: 1) patients undergoing spine surgery; 2) comparing TLIP block and/or modified TLIP block with no block care or wound infiltration 3) pain or morphine consumption as a primary or secondary outcome; 4) randomized controlled trials. We excluded studies if they 1) had other additional treatment in experimental or control group; 2) were not able to extract data; 3) were not available for full text.

### Method of review

Each article was reviewed by 2 independent researchers who use the double-extraction method for meeting our inclusion criteria, then it was confirmed by a third reviewer, all the disagreement was resolved before the final analysis. 2 researchers were in charge of the data extraction work. The following information was recorded: the first author, the publication time, study design, study name, participant characteristics, outcome measures, surgical procedure, time of follow-up, pain score [time, mean, and standard deviation (SD)], local anesthetic administration characteristics, postoperative analgesic administration, use of ultrasound guidance and endpoints of each study. Numerical rating scale of pain or visual analog scale was adapted to an 11-point numeric rating scale (0 = no pain, 10 = extreme pain). Postoperative opioids were transformed to an equianalgesic dose of intravenous morphine assuming no cross-tolerance. The quality of the RCTs was assessed with the use of the Cochrane Collaboration’s recommended tool by 2 reviewers [13]. 7 potential risks of bias were judged according to the assessment tool: random sequence generation, allocation concealment, blinding of participants, blinding of outcome assessment, incomplete outcome data, selective reporting and other bias. The assessment of each domain was rated either as low risk, high risk or unclear risk. Disagreement were solved through discussion.

The primary outcome was cumulative morphine consumption during the 0-24-hour postoperative period. The secondary endpoints were defined as patients’ self-reported level of pain intensity with rest or movement on 0–10 pain scales such as visual analog scales (VAS), numerical rating scales (NRS), and other validated pain scales in the postoperative period (1–2 hours, 2–4 hours, 4–8 hours, 8–12 hours, 24 hours after surgery), rescue analgesia requirement, the adverse events associated with the use of TLIP block such as postoperative nausea and vomiting (PONV), block failure, and block-related complications such as neuraxial injury.

### Statistical analysis

Continuous data were analyzed using weight mean differences (WMDs) or standard mean differences (SMDs) and their 95%CIs for combining various scales. Data provided as mean and standard deviation were extracted. Data provided as standard error were transformed to standard deviation (SD) through the formula: SD = SE*√n (n = sample size). Data provided as median and interquartile range (IQR) was transformed to standard deviation through the formula: SD = IQR/1.349. Data provided as the confidence interval was transformed to standard deviation through the formula: 95%CI = x±1.96*SE where SD = SE*√(n). Data provided as median and the range was transformed to standard deviation based on the sample size through the formula described by Hozo et al [14]. When the same outcomes such as visual analog scales (VAS), numerical rating scales (NRS) were reported more than once, the most conservative value was used. For dichotomous data, relative risks (RR) with 95%CI were estimated. The heterogeneity among these included studies was evaluated by I^2^ statistics, random effect model was applied when there was high heterogeneity (I^2^>30%) across the studies, whereas significant heterogeneity was not observed across the studies(I^2^≤30%), fixed effect model was applied. Publication bias was examined by the Egger test. All statistical was executed by stata15.1. Statistical significance was represented by p<0.05.

### Ethics approval and consent to participate

No patients or members of public were involved in the present study. No patients were asked to advise on the interpretation or writing up of results. The results of the present research will be communicated to the relevant patient community.

## Result

**Fig 1:**
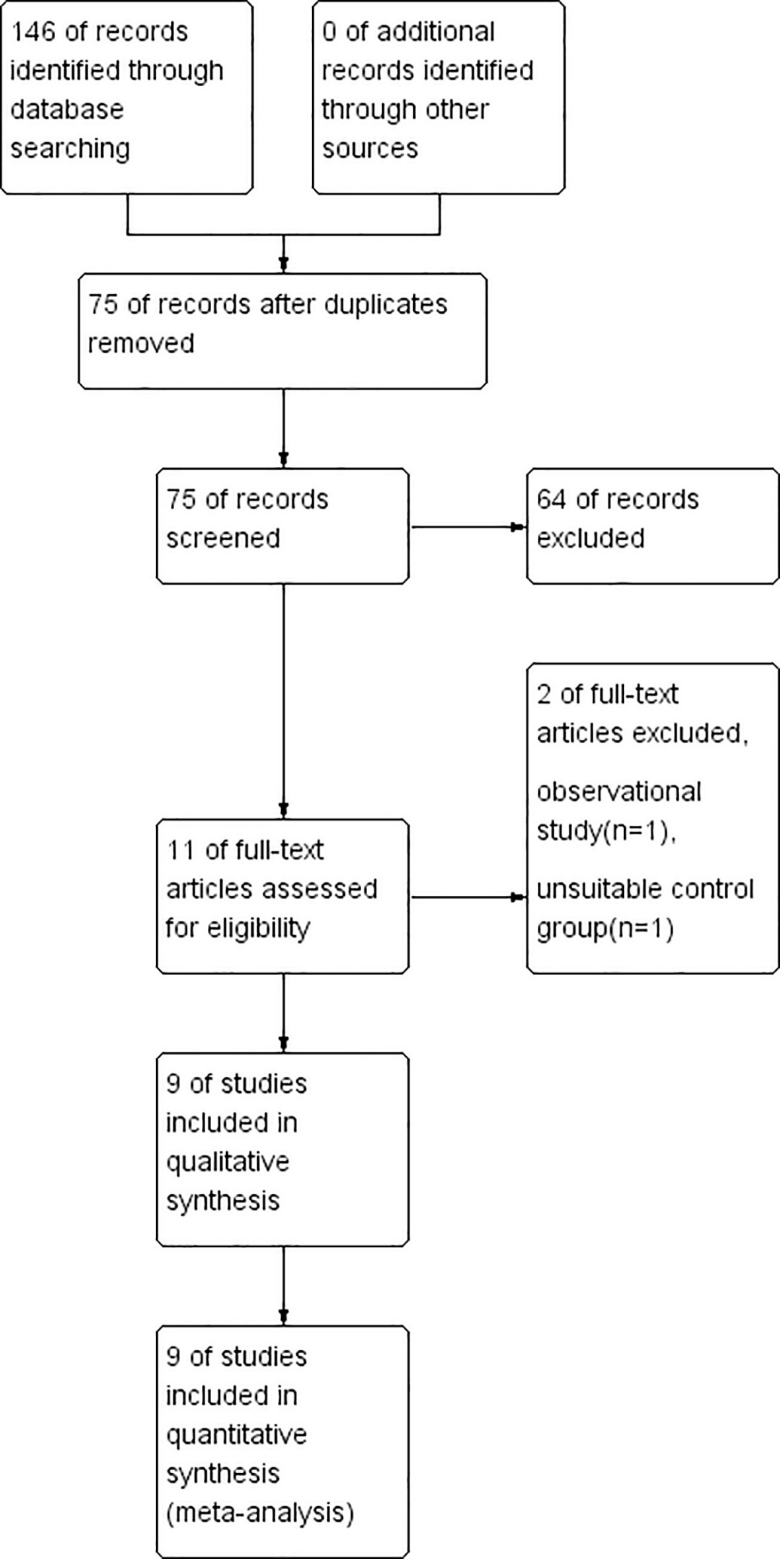
PRISMA flow diagram.

**Fig 2:**
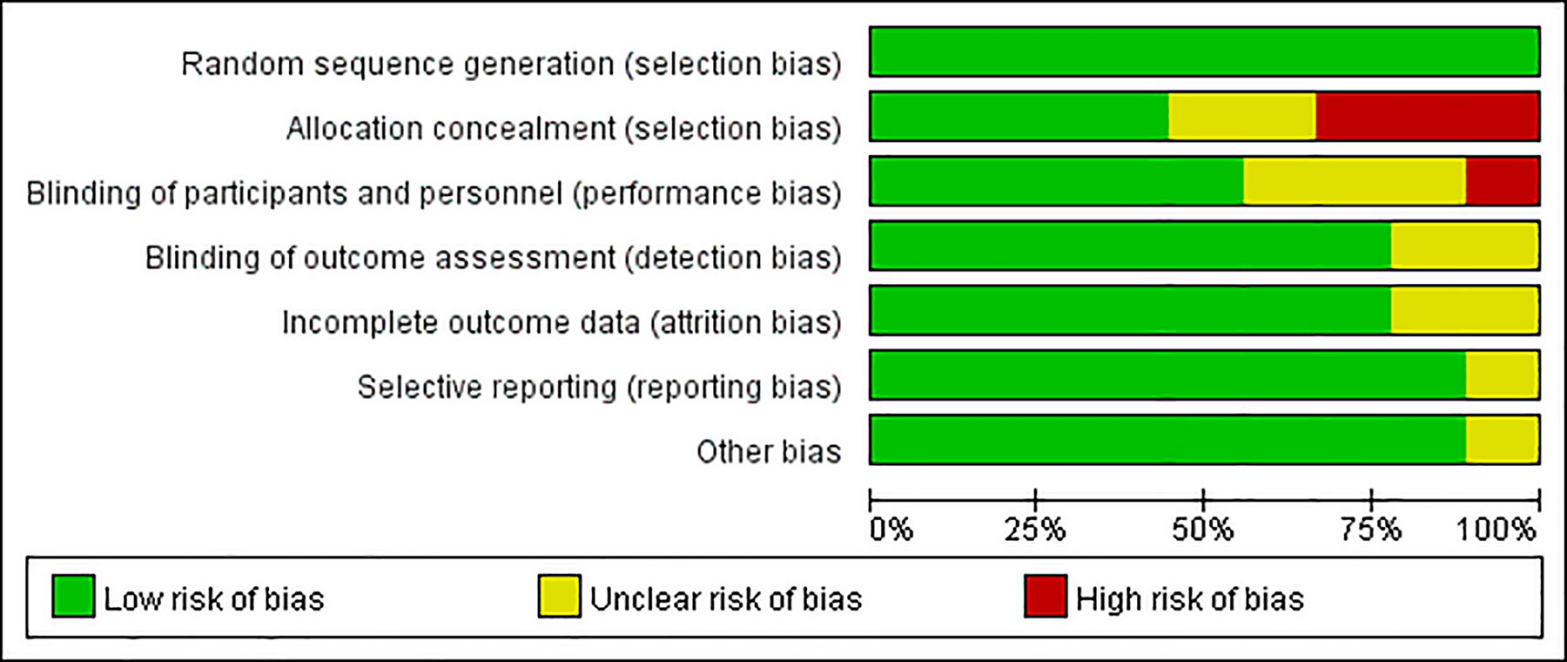
Risk of bias graph.

**Fig 3:**
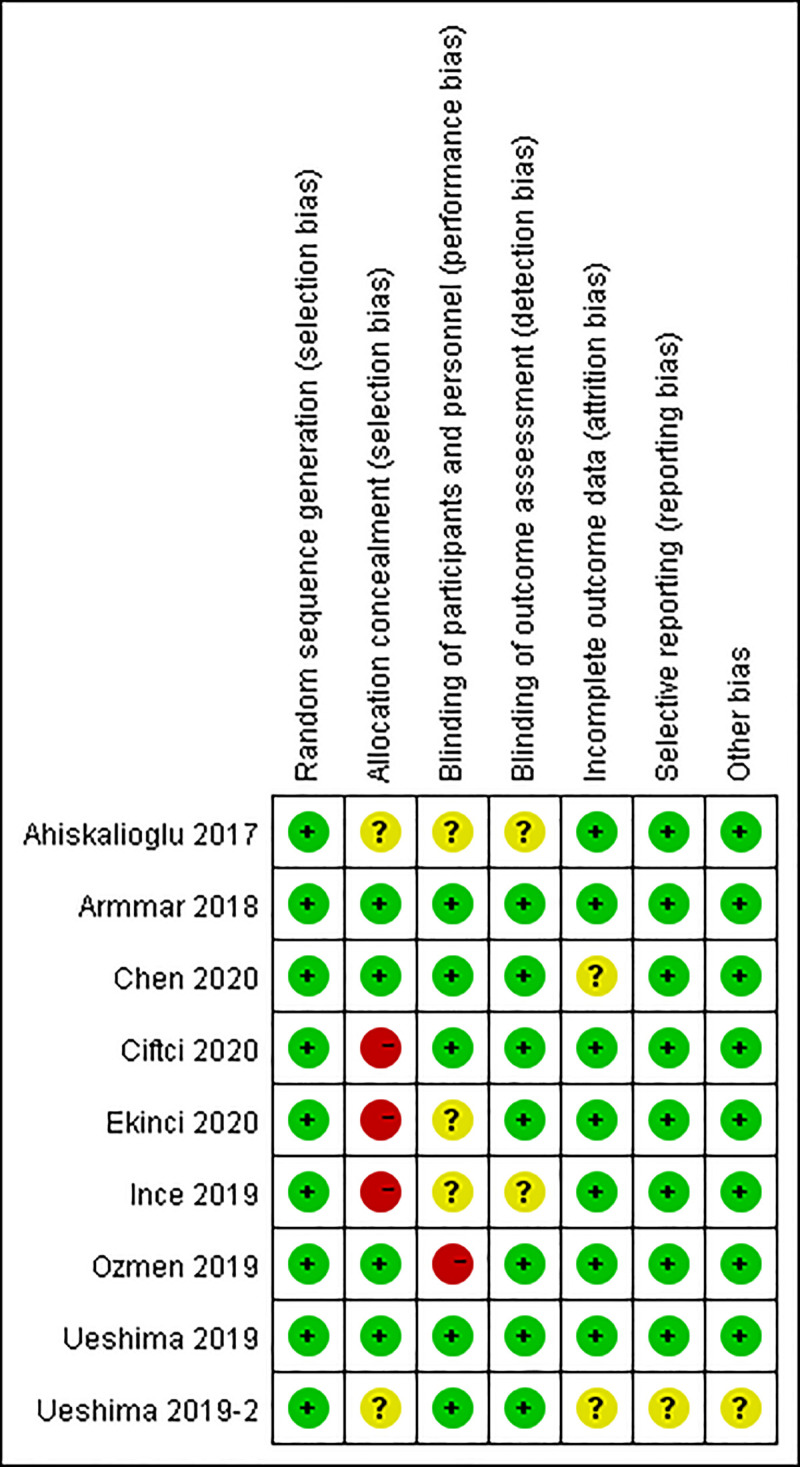
Risk of bias summary.

We identified 75 studies in the initial literature research. Based on the inclusion criteria, 64 studies were excluded, with a selection of 11 studies for a more detailed review. 2 studies were subsequently excluded, leaving 9 randomized controlled trials (Fig 1). Finally, 9 randomized controlled trials comprising 539 patients were included for meta-analysis [10–12, 15–20]. The characteristics of included studies are presented in Table 1. The methodological quality of the involved trials is shown in Figs 2 and 3. 6 studies compared TLIP block with non-block care [10, 12, 15, 16, 18, 20], 3 studies compared TLIP block with wound infiltration [11, 17, 19].

**Table 1:** Characteristics of included studies.

Author	Year	Procedure	USG	Number of patients	Block/ control	Dose (for each side)	Primary outcome	Type of study
Ahiskalioglu et al.	2017	Spinal surgery	Y	40	modified TLIP(bupivacaine)+GA versus TLIP(NS)+GA	20 ml 0.25% bupivacaine	Fentanyl consumption durning 24 hours	RCT
Ueshima et al.	2019	Primary lumbar lamino plasty of less than 3 levels	Y	69	classical TLIP(bupivacaine)+GA versus TLIP(NS)+GA	20 ml 0.25% bupivacaine	Fentanyl consumption durning 48 hours	RCT
Ekinci et al.	2020	Lumbar spinal surgery	Y	60	modified TLIP(bupivacaine)+GA versus wound infiltration+GA	20 ml 0.25% bupivacaine	Opioid consumption durning 24 hours	RCT
Ince et al.	2019	Single-level discectomy	Y	40	classical TLIP(bupivacaine)+GA versus wound infiltration+GA	20 ml 0.25% bupivacaine	Opioid consumption durning 24 hours	RCT
Ozmen et al.	2019	Single-level discectomy	Y	80	modified TLIP(bupivacaine)+GA versus TLIP(NS)+GA	20 ml 0.25% bupivacaine	QoT-40 scores	RCT
Chen et al.	2019	Lumbar spine fusion surgery	Y	60	classical TLIP(bupivacaine)+GA versus TLIP(NS)+GA	20 ml 0.25% bupivacaine	Perioperative opioid consumption	RCT
Ueshima et al.	2019	Lumbar spinal surgery	Y	60	classical TLIP(bupivacaine)+GA versus wound infiltration+GA	20 ml 0.375% bupivacaine	Cumulative fentanyl administered for rescue analgesia	RCT
Armmar et al.	2018	Herniated lumbar disc surgery	Y	70	classical TLIP(bupivacaine)+GA versus no block+GA	20 ml mixture of 0.25% bupivacaine and 1% lidocaine	VAS scores	RCT
Ciftci et al.	2020	Lumbar Discectomy Surgery	Y	60	Modified TLIP(bupivacaine)+GA versus no block+GA	20 ml 0.25% bupivacaine	Fentanyl consumption durning 24 hours	RCT

TLIP: thoracolumbar plane block, USG: ultra-sound guided, GA: general anesthesia, NS: natural saline, VAS: visual analog scales, RCT: randomized controlled trials.

### Cumulative morphine consumption

**Fig 4:**
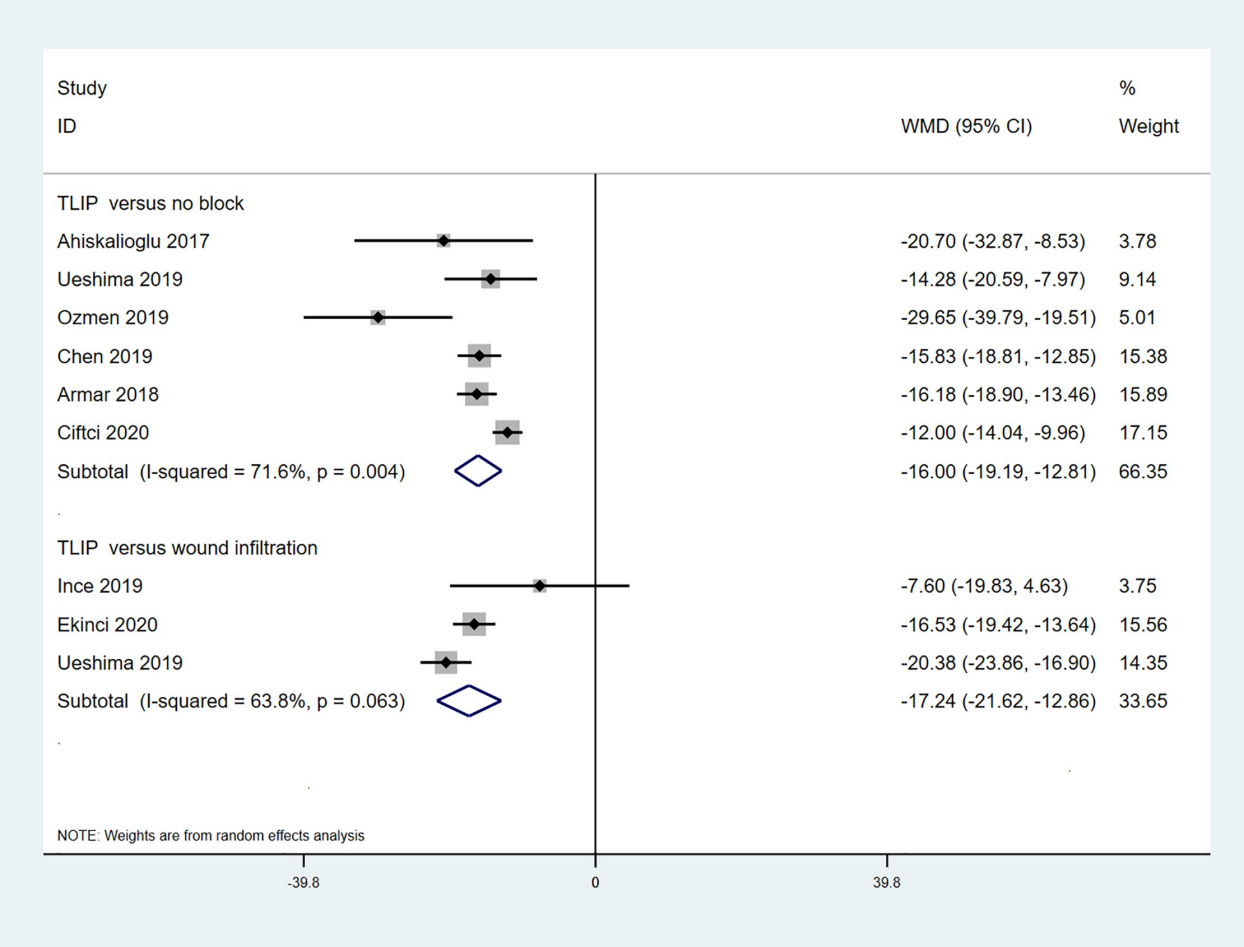
Forest plot for the comparison of morphine equivalents (mg) in the first 24 h after surgery.

9 studies reported the cumulative morphine consumption during 0-24-hour periods (Fig 4) [10–12, 15–20]. The pooled data from 6 studies showed that TLIP block reduced the cumulative morphine consumption significantly when compared with non-block group (WMD -16.00; 95%CI -19.19, -12.81; p<0.001; I^2^ = 71.6%) [10, 12, 15, 16, 18, 20]. The pooled data from 3 studies showed that TLIP block reduced the cumulative morphine consumption significantly compared with wound infiltration group (WMD -17.23, 95%CI -21.62, -12.86; p<0.001; I^2^ = 63.8%) [11, 17, 19]. Result of the Egger test suggested that any publication bias across included studies was unlikely (TLIP block versus non-block: p = 0.241, TLIP block versus wound infiltration: p = 0.615).

### Postoperative pain intensity

Across all studies, 8 studies were analyzed postoperative pain intensity with the use of TLIP block in patients receiving spine surgery [10–12, 15–18].

**Fig 5:**
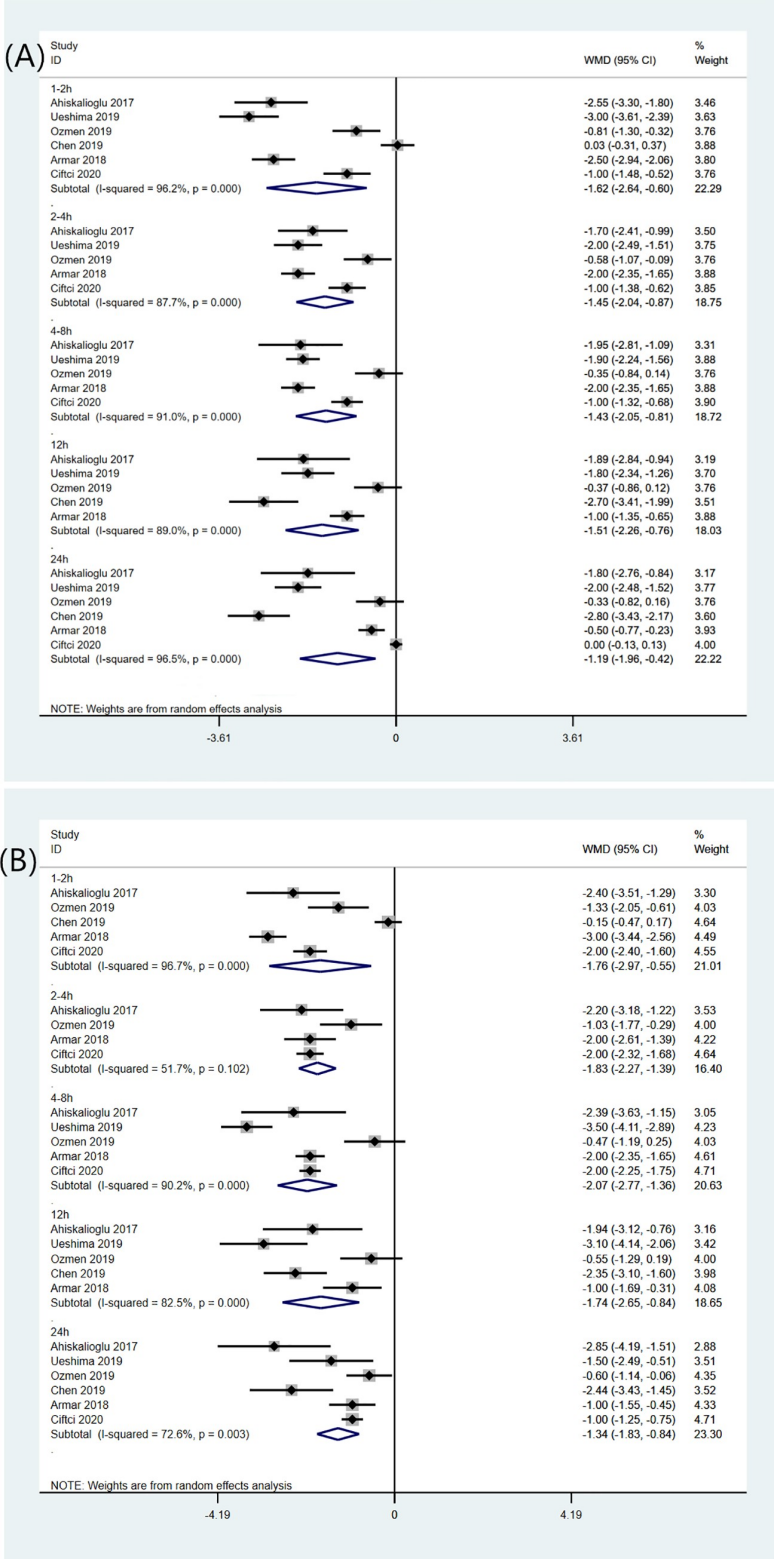
Forest plot of pain intensity for the TLIP block versus non-block care studies in the first 24 h after surgery. (A) Pain intensity at rest for the TLIP block versus non-block care studies in the first 24 h after surgery. (B) Pain intensity at movement for the TLIP block versus non-block care studies in the first 24 h after surgery.

6 studies compared the postoperative pain intensity at rest between patients receiving TLIP block and non-block care. The TLIP block significantly reduced pain intensity at rest at all time points postoperatively compared with non-block care group (Fig 5A) [10, 12, 15, 16, 18, 20]: at 1–2 h (WMD -1.62; 95%CI -2.64, -0.60; p = 0.002; I^2^ = 96.2%); at 2-4h (WMD -1.45; 95%CI -2.04, -0.87; p<0.001; I^2^ = 87.7%); at 4-8h (WMD -1.43; 95%CI -2.05, -0.81; p<0.001; I^2^ = 91.0%); at 12h (WMD -1.51; 95%CI -2.26,-0.76; p<0.001; I^2^ = 89.0%); at 24h (WMD -1.19; 95%CI -1.96, -0.42; p = 0.002; I^2^ = 96.5%). The TLIP block significantly reduced pain scores at movement at all time points postoperatively compared with non-block care group (Fig 5B) [10, 12, 15, 16, 18, 20]: at 1-2h (WMD -1.76; 95%CI -2.97, -0.55; p = 0.004; I^2^ = 96.7%); at 2-4h (WMD -1.83; 95%CI -2.27, -1.39; p<0.001; I^2^ = 51.7%); at 4-8h (WMD -2.07; 95%CI -2.77, -1.36; p<0.001; I^2^ = 90.2%); at 12h (WMD -1.74; 95%CI -2.65, -0.84; p<0.001; I^2^ = 82.5%); at 24h (WMD -1.34; 95%CI -1.83, -0.84; p = 0.001; I^2^ = 72.6%). A sensitivity analysis by removing individual studies did not significantly reduce heterogeneity. Besides, one study reported that patients receiving TLIP block had better quality of recovery in the POD1 compared with non-block group [10].

**Fig 6:**
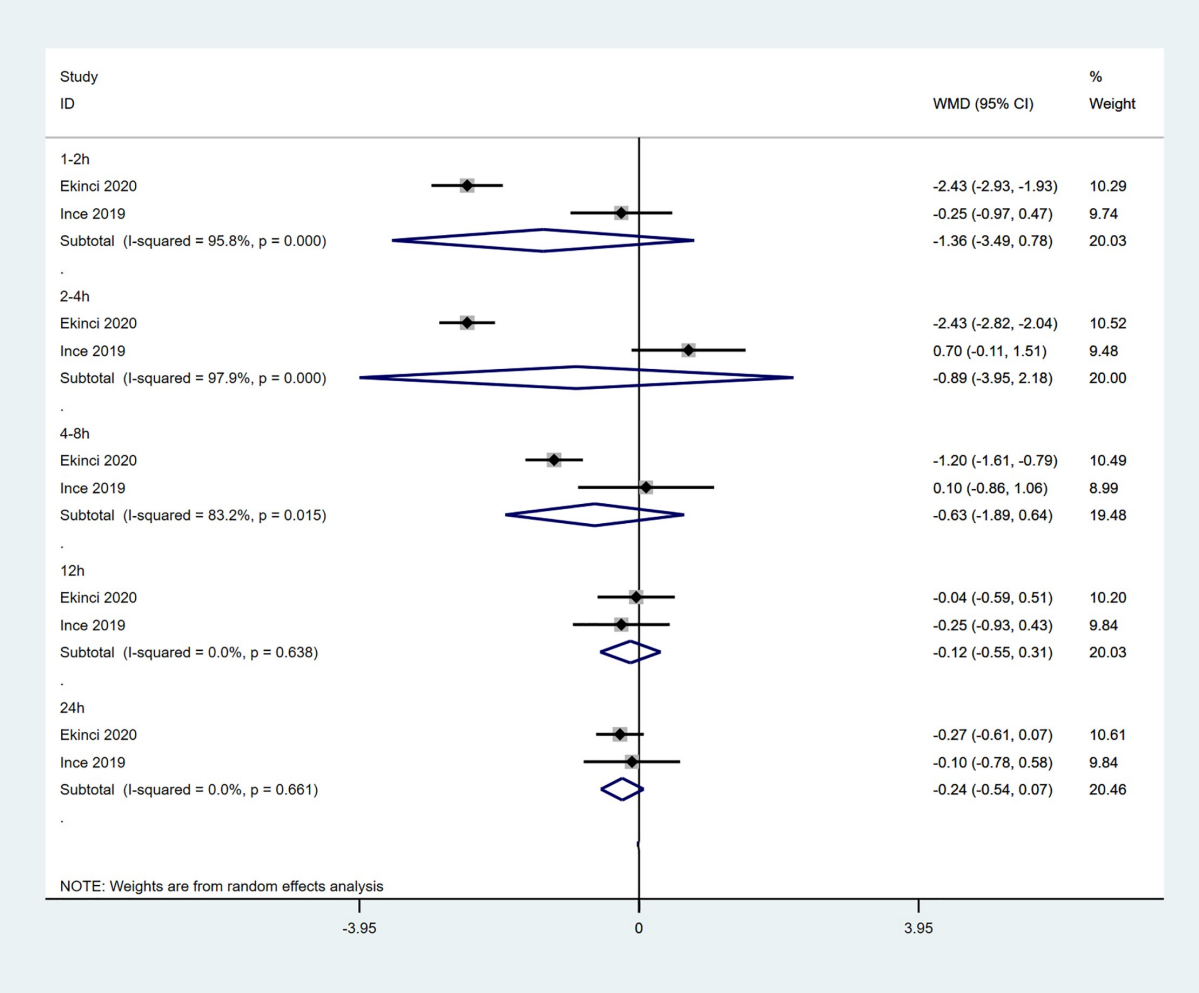
Forest plot of pain intensity at rest for the TLIP block versus wound infiltration studies in the first 24 h after surgery.

2 studies compared the postoperative pain intensity at rest between patients receiving TLIP block and wound infiltration (Fig 6) [11, 17]. There was no significant difference between these 2 groups on meta-analysis pain intensity at rest at all time points postoperatively: at 1-2h (WMD -1.36; 95%CI -3.49, 0.78; p = 0.214; I^2^ = 95.8%); at 2-4h (WMD -0.89; 95%CI -3.95,2.18; p = 0.571, I^2^ = 97.9%); at 4-8h (WMD -0.63; 95%CI -1.89, 0.64; p = 0.332; I^2^ = 83.2%); at 12h (WMD -0.12; 95%CI -0.55, 0.31; p = 0.577; I^2^ = 0%); at 24h (WMD -0.65; 95%CI -0.54, 0.07; p = 0.124; I^2^ = 0.0%). 1 study reported patients receiving TLIP block had lower postoperative pain intensity at movement compared with patients receiving wound infiltration in the first 8 hours after surgery, but the difference was not significant at later time points [11].

### Rescue analgesic requirement

**Fig 7:**
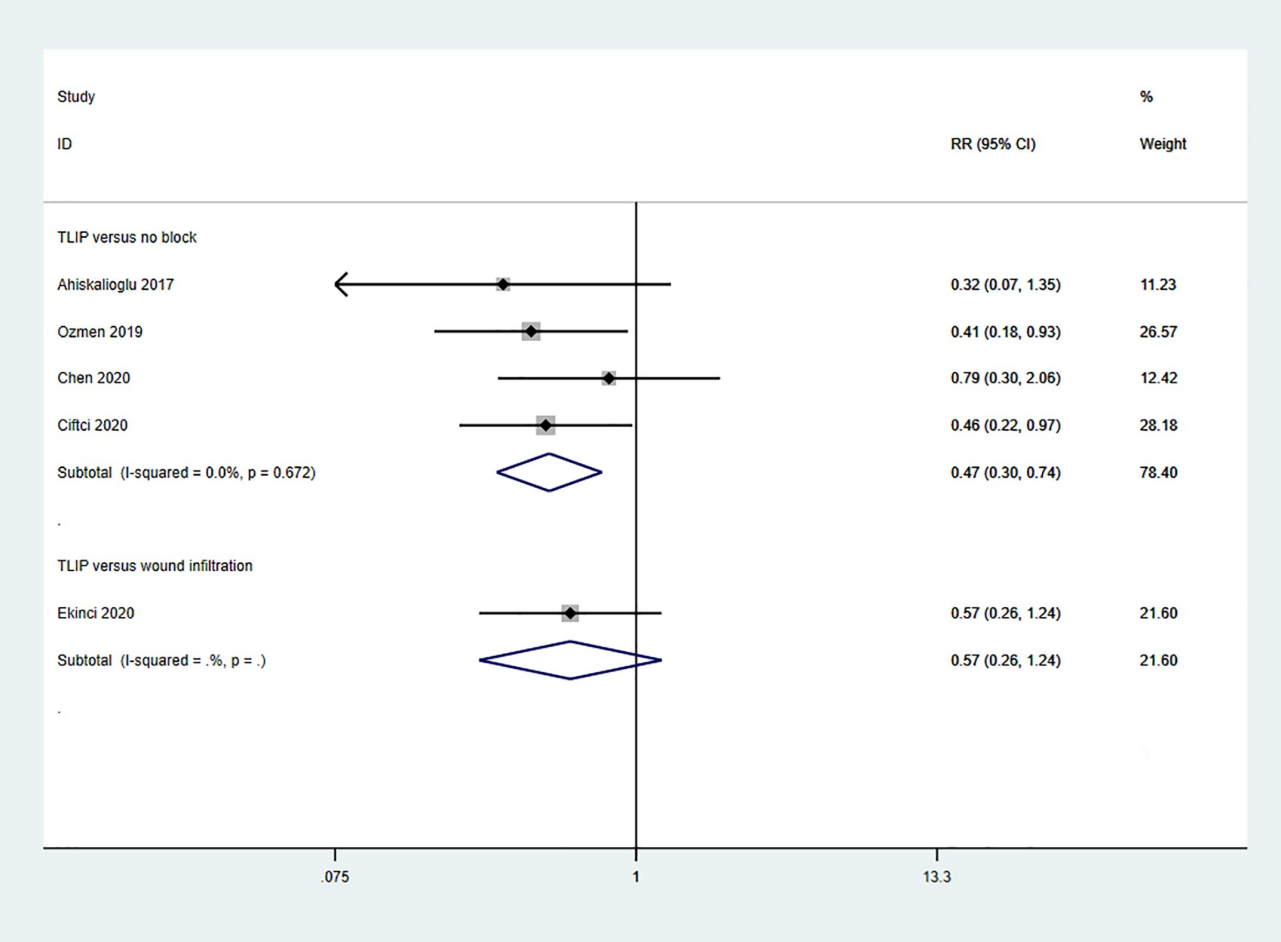
Forest plot of the incidence of rescue analgesia requirement.

5 studies reported the incidence of rescue analgesia requirement (Fig 7) [10–12, 15, 20]. The pooled data from 4 studies showed that TLIP block reduced the incidence of rescue analgesia requirement significantly compared with non-block group (RR 0.47; 95%CI 0.30, 0.74; p = 0.001; I^2^ = 0.0%). 1 study reported that patients receiving TLIP block had less incidence of rescue analgesia requirement than patients receiving wound infiltration, but it was not statistically significant (RR 0.57;95%CI 0.26, 1.24; p = 0.157).

### Adverse events and TLIP block-related complications

**Fig 8:**
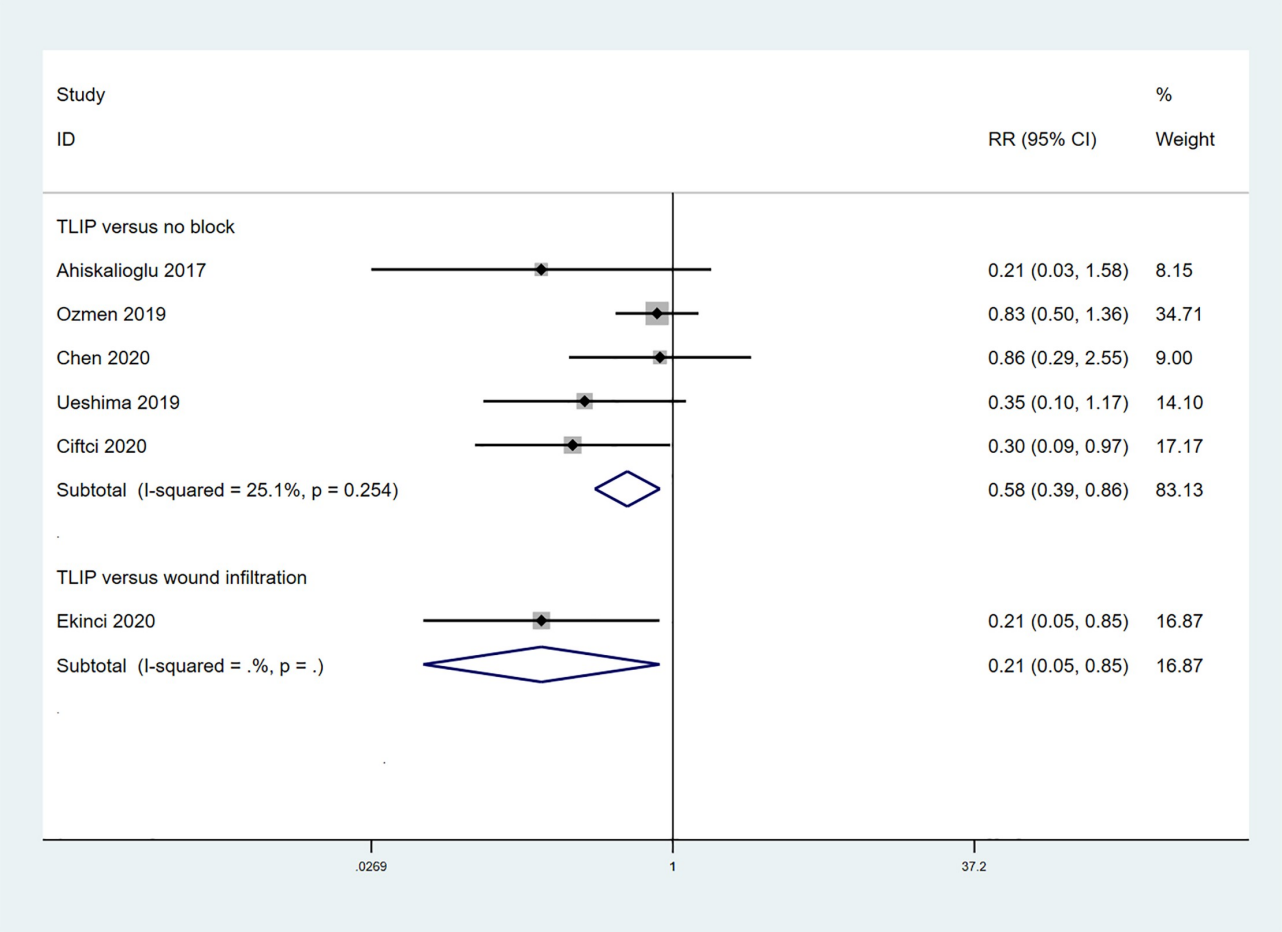
Forest plot of the incidence of PONV.

6 studies assessed the impact of TLIP block on the incidence of PONV in patients undergoing spine surgery (Fig 8) [10–12, 15, 18, 20]. The pooled data from 5 studies showed that TLIP block significantly reduced the incidence of PONV compared to the non-block group (RR 0.58; 95%CI 0.39, 0.86; p = 0.006; I^2^ = 25.1%). 1 study reported that patients receiving TLIP block had less incidence of PONV than patients receiving wound infiltration (RR 0.21; 95%CI 0.05, 0.852; p = 0.029).

One study reported 2 patients experienced unsuccessful TLIP block, this would translate to an incidence (95%CI) of 0.8% (0.3%, 2%) when considering the aggregate number of patients [10]. There has been no major adverse event and complication related to TLIP block and wound infiltration according to our systematic review, such as local anesthetic systemic toxicity, neuraxial injury, hematoma, and puncture site infection.

## Discussion

The current evidence showed that TLIP block is clinically superior to non-block care with regard to the cumulative opioid consumption, acute pain intensity, rescue analgesia requirement, and PONV in the postoperative period for patients undergoing spine surgery. Besides, TLIP block also reduced the cumulative opioid consumption compared to wound infiltration, but the postoperative pain intensity at rest was comparable during the first 24h after surgery between TLIP block and wound infiltration, only limited evidence showed that TLIP block attenuated pain intensity at movement during the first 8h after surgery and reduced the incidence of PONV and rescue analgesia requirement, more quantitative studies comparing these regional anesthesia techniques are needed to confirm the findings.

Our results are of clinical importance, because spine surgery is extremely painful and the postoperative pain is usually hard to control, besides, and massive use of opioids is associated with adverse clinical events [21, 22]. The uncontrolled acute postoperative pain could promote the development of chronic persistent pain, which could worsen their independence, mood, and quality of life [23–25]. Hence, there is a possibility that TLIP block could not only attenuate the acute pain after surgery, but reduce the development of chronic persistent pain, further studies with longer follow-up time are needed to test the long-term benefit.

Our meta-analysis found statistically significant difference in PONV and rescue analgesia requirement between the TLIP block and non-block groups. Additionally, according to our systematic review, we found no major adverse events and complication related to the TLIP block. Current evidence showed that TLIP block is a promising regional anesthesia technique for spine surgery.

TLIP block is a type of interfascial plane block that targets the dorsal rami of the thoracolumbar nerves, in classic TLIP block, the local anesthetic was injected between the multifidus and longissimus muscles by advancing the needle from lateral to medial side [7]. Due to the risk of accidental neuraxial anesthesia and the difficulties of sonographic image between multifidus and longissimus muscles, the modified TLIP block was described in 2017, in modified TLIP block, the local anesthetic was injected between longissimus and iliocostalis muscles by advancing the needle from medial to lateral [8]. It is reported in a randomized controlled study that modified TLIP block had shorted performance time, higher success rate of one-time block but similar analgesia effect compared with classic TLIP block [9].

Wound infiltration has been reported to barely reduce the opioid consumption several times, because its analgesia effect depends on the absorption of the local anesthetic [11, 26, 27]. Wound infiltration is an easy and simple technique to perform, but the local anesthetic was injected blindly into the wound, and was effective only at the administration site [11]. We found that TLIP block was superior to wound infiltration in terms of opioid consumption in our study.

There were also alternative regional anesthesia techniques that target the ventral rami such as retrolaminar block and erector spine block being reported to have analgesia effect after spine surgery [18, 28–31]. However, there was no evidence that compared the alternative regional anesthesia techniques with TLIP block about efficacy and safety until now. TLIP block has shallower injection site compared to the above anesthesia techniques, so the time to treat complications such as hematoma might be shorter in TLIP block [18]. Besides, intraoperative spinal cord monitoring including somatosensory evoked potentials (SEP) and motor evoked potentials (MEP) also limits the use of the alternative anesthesia techniques, as the nerves monitor stem from ventral rami [32, 33]. Moreover, spine surgery often uses a posterior midline incision, which is innervated by the dorsal ram [33].i Further studies are needed to compare the efficacy and safety of these regional anesthesia techniques and find out the best one for the patients undergoing spine surgery.

Our study was also inevitable in shortage. First, significant heterogeneity was observed in some of our results, although sensitivity analyses were done, we did not identify the source of heterogeneity. Second, we did not examine a dose-response effect of the TLIP block as the dosage used in the included studies did not provide enough variability. Third, there was significant inter-study difference in the way of reporting opioid dosages and pain scores, the diversity in the pain intensity assessment tools (VRS, NRS, VAS) and follow-up time might lead to heterogeneity and deviation in the analysis. Fourth, the sample size of included studies was relatively small, with the largest study only including 80 patients, Fifth, few studies reported the length of hospital stay, the time to first analgesia request, length of PACU stay, future studies should perform the analysis of TLIP block by using the above data.

## Supporting information

S1 Checklist PRISMA 2009 checklist. (DOC)

S1 Data (XLSX)

S1 Table Characteristics of included studies. (DOCX)

## Abbreviations

NSAIDs: Non-steroid anti-inflammatory drugs
TLIP block: thoracolumbar interfascial block
SD: standard deviation
VAS: visual analog scales
NRS: numerical rating scales
WMDs: weight mean differences
SMDs: standard mean differences
IQR: interquartile range
CI: confidence interval
PONV: postoperative nausea and vomiting
USG: ultra-sound guided
