# Supplementary material for: Thoracolumbar interfascial plane block for postoperative analgesia in spine surgery: A systematic review and meta-analysis
Source: PLoS One. 2021 May 21;16(5):e0251980. doi: 10.1371/journal.pone.0251980 (PMC8139495; doi:10.1371/journal.pone.0251980)
Supplement: S1 Table — (DOCX) [file pone.0251980.s003.docx]

| S1 Table. Characteristics of included studies | | | | | | | | |
| --- | --- | --- | --- | --- | --- | --- | --- | --- |
| Author | Year | Procedure | USG | Number of patients | Block/ control | Dose (for each side) | Primary outcome | Type of study |
| Ahiskalioglu et al. | 2017 | Spinal surgery | Y | 40 | modified TLIP(bupivacaine)+GA versus TLIP(NS)+GA | 20 ml 0.25% bupivacaine | Fentanyl consumption durning 24 hours | RCT |
| Ueshima et al. | 2019 | Primary lumbar lamino plasty of less than 3 levels | Y | 69 | classical TLIP(bupivacaine)+GA versus TLIP(NS)+GA | 20 ml 0.25% bupivacaine | Fentanyl consumption durning 48 hours | RCT |
| Ekinci et al. | 2020 | Lumbar spinal surgery | Y | 60 | modified TLIP(bupivacaine)+GA versus wound infiltration+GA | 20 ml 0.25% bupivacaine | Opioid consumption durning 24 hours | RCT |
| Ince et al. | 2019 | Single-level discectomy | Y | 40 | classical TLIP(bupivacaine)+GA versus wound infiltration+GA | 20 ml 0.25% bupivacaine | Opioid consumption durning 24 hours | RCT |
| Ozmen et al. | 2019 | Single-level discectomy | Y | 80 | modified TLIP(bupivacaine)+GA versus TLIP(NS)+GA | 20 ml 0.25% bupivacaine | QoT-40 scores | RCT |
| Chen et al. | 2019 | Lumbar spine fusion surgery | Y | 60 | classical TLIP(bupivacaine)+GA versus TLIP(NS)+GA | 20 ml 0.25% bupivacaine | Perioperative opioid consumption | RCT |
| Ueshima et al. | 2019 | Lumbar spinal surgery | Y | 60 | classical TLIP(bupivacaine)+GA versus wound infiltration+GA | 20 ml 0.375% bupivacaine | Cumulative fentanyl administered for rescue analgesia | RCT |
| Armmar et al. | 2018 | Herniated lumbar disc surgery | Y | 70 | classical TLIP(bupivacaine)+GA versus no block+GA | 20 ml mixture of 0.25% bupivacaine and 1% lidocaine | VAS scores | RCT |
| Ciftci et al. | 2020 | Lumbar Discectomy Surgery | Y | 60 | Modified TLIP(bupivacaine)+GA versus no block+GA | 20 ml 0.25% bupivacaine | Fentanyl consumption durning 24 hours | RCT |
| TLIP: thoracolumbar plane block, USG: ultra-sound guided, GA: general anesthesia, NS: natural saline, VAS:visual analog scales, RCT: randomized controlled trials | | | | | | | |  |
